# Employment trajectories and mental health-related disability in Belgium

**DOI:** 10.1007/s00420-022-01923-y

**Published:** 2022-10-10

**Authors:** Rebeka Balogh, Sylvie Gadeyne, Johanna Jonsson, Sudipa Sarkar, Karen Van Aerden, Chris Warhurst, Christophe Vanroelen

**Affiliations:** 1 Interface Demography, Department of Sociology, Vrije Universiteit Brussel, Pleinlaan 2, 1050 Brussels, Belgium; 2 Institute for Employment Research, University of Warwick, Coventry, UK; 3 Unit of Occupational Medicine, Institute of Environmental Medicine, Karolinska Institutet, Stockholm, Sweden; 4 Institute of Public Policy, National Law School of India University, Bengaluru, India

**Keywords:** Employment trajectory, Mental health, Disability, Employment quality, Belgium, Precarious employment

## Abstract

**Objectives:**

An individual’s quality of employment over time has been highlighted as a potential determinant of mental health. With mental ill-health greatly contributing to work incapacities and disabilities in Belgium, the present study aims to explore whether mental health, as indicated by registered mental health-related disability, is structured along the lines of employment quality, whereby employment quality is assessed over time as part of individuals’ labour market trajectories.

**Methods:**

Using administrative data from the Belgian Crossroads Bank for Social Security over 16 quarters between 2006 and 2009, transitions between waged jobs of varying quality (based on dimensions of income, working time, employment stability and multiple jobholding), self-employment, and unemployment are considered among individuals in the labour force aged 30–40 at baseline (*n* = 41,065 women and 45,667 men). With Multichannel Sequence Analysis and clustering, we constructed ideal types of employment trajectories. Fitting Cox regressions, we then evaluated individuals’ hazard of experiencing a disability from a mental disorder between 2010 and 2016.

**Results:**

Our analysis highlights various gender-specific trajectories. Among both genders, individuals exposed to near-constant unemployment over the initial 4 years showed the highest hazard of subsequent mental health-related disability compared to a group characterised by stable full-time employment, single jobholding, and above-median income. Trajectories involving a higher probability of subsidised and non-standard employment and (potential) spells of unemployment and lower relative income were also strong predictors of cause-specific disabilities. Health selection and confounding might, however, be contributing factors.

**Conclusions:**

Our study shows a gradient of mental disorders resulting in a disability along trajectory types. Our findings highlight the predictive power of labour market trajectories and their employment quality for subsequent mental disorder-related disability. Future research should examine the mechanisms, including selection effects in this association.

**Supplementary Information:**

The online version contains supplementary material available at 10.1007/s00420-022-01923-y.

## Introduction

Historically, concerns have been raised about the quality of work and employment that comprise jobs (e.g.,Braverman 1998; Quinlan 2012). Recent changes to labour markets in North America and Western Europe have re-focused research and policy attention onto the quality of jobs (Kalleberg 2000, 2011; Kalleberg and Vallas 2018). This shift ended what Kalleberg (2011, p. 21) and Quinlan (2012, p. 19) call the post-war “interregnum”, a period characterised by what has been termed the ‘standard employment relationship’ (henceforth SER): full-time employment marked by security and predictability and underpinned by a particular ‘settlement’ between capital and labour (see Bosch 2004; Vosko 2006). From the 1970s, a shift in government economic policy, transformation in the composition of the labour force, deregulation of finance and labour markets and a shrinking of union power shifted the balance of power between labour and capital toward the latter and led to a “resurgence” of poorer quality employment arrangements, weakening the SER (Kalleberg and Vallas 2018, p. 6; see also Kalleberg 2011; Vosko et al. 2009). Nonetheless, the impact of the SER can still be felt: it established a strong ‘mental frame’ which still serves as a point of reference to contrast lower quality employment arrangements (Vosko et al. 2009). In particular, non-standard forms of employment (including temporary agency, seasonal and part-time) have gained prominence in recent years (Eurofound 2018; International Labour Office 2016; Kalleberg 2000). Arguments have been made, however, that focusing solely on the type of employment contract might limit understanding of the true nature of non-standard employment (Rodgers 1989).

The concept of ‘precarious employment’ evolved as a result. This concept encapsulates an employment form that is insecure (Kalleberg and Vallas 2018; Rodgers 1989; Vosko 2006), is linked to reduced social benefits (Kalleberg and Vallas 2018; Vosko 2006), and associated with low income or wages (Rodgers 1989; Vosko 2006), is potentially harmful to workers’ health (Vosko 2006) and, as such, is best understood multidimensionally (Kalleberg and Vallas 2018; Rodgers 1989; Vosko 2006). Concerned about its potential health implications, various scales of employment precariousness have been developed in recent years to measure and monitor this concept (Jonsson et al. 2021a; Padrosa et al. 2021; Vives et al. 2010). In addition, and analogous to developments in the study of job quality (Holman 2013; Wright et al. 2018), a more holistic way of understanding various employment forms evolved along the spectrum of *employment quality* (Vanroelen 2019). Employment quality refers to the conditions of and relations surrounding an employment arrangement (Van Aerden et al. 2014; Vanroelen 2019), which, along with the organisation and nature of the work framed by the terms of employment, constitute job quality (Warhurst et al. 2017). Precarious employment can then be conceived as a cluster of arrangements situated at the lower end of the employment quality distribution, strongly diverging from the ideal type of SER; other employment types, such as ‘portfolio’ jobs, have also been identified as constituting an arrangement of different employment qualities (Van Aerden et al. 2014, 2016; Jonsson et al. 2021a; Peckham et al. 2019). In sum, existing research reveals a typology of employment arrangements of varying employment quality, which are in turn clearly related to a gradient of mental (and general) health across the working population (Gevaert et al. 2021; Peckham et al. 2019; Van Aerden et al. 2016; Peckham et al. 2019).

### Mental health and the quality of employment: from cross-sectional to longitudinal typologies

Mental health conditions are one of the leading causes of (increasing) work incapacities in Belgium (Plasman et al. 2015). Examining the global burden of diseases also reveals that major depressive disorder contributed greatly to the number of years lived with disability in the country in 2016 (Maertens De Noordhout et al. 2018). At the same time, good mental health is not distributed equally among the labour force. While psychosocial factors are important work-related determinants of mental health (Stansfeld and Candy 2006), studies from Spain, the US and across the EU also reveal inequalities along the lines of employment conditions and relations (Peckham et al. 2019; Vives et al. 2011; Van Aerden et al. 2016). Moreover, a mental health gap exists between the employed and the unemployed in terms of scarring effects (Strandh et al. 2014). While having a strong collective bargaining system and established employment rights (Vandaele 2019), the quality of employment in Belgium is a concern from a health perspective. Non-standard employment (especially among men) has been linked to elevated of mortality over a 13-year period (Balogh et al. 2021), and evidence has also emerged that those in more unstable and lower-income (‘precarious’) as well as secure jobs with fewer benefits (‘instrumental jobs’) report worse mental health than their counterparts in standard employment (Van Aerden et al. 2017). What is now needed is analysis that explores whether these inequalities also translate into diagnosed mental ill-health resulting in an unequal distribution of work incapacity and disability along the lines of employment quality.

At the same time, there is an urgent need to shift the perspective from ‘good’ (quality) and ‘bad’ (quality) *employment* to ‘good’ and ‘bad’ (quality) *employment trajectories*, particularly when it comes to studying work-related health implications. This need arises, because working lives are not static. For many workers, an employment trajectory involves moving in and out of employment as well as moving between different employment arrangements, both of which cross-sectional studies fail to capture. Consequently, while there is evidence that workers clustering around the lower end of employment quality tend to have worse mental health than those in more standard employment arrangements (Peckham et al. 2019; Van Aerden et al. 2016, see above), little is known on whether or to what extent this link can be ascribed to prior experience of unemployment (Benach et al. 2014), or to what extent this association reflects a previously accumulated health disadvantage (see also Amick et al. 2016). Improvements in data availability and methodological advancements focusing on the life-course—such as Sequence Analysis (Abbott 1995)—are beginning to enable much-needed longitudinal empirical analyses mapping long-term trajectories and looking at links to individuals’ health. Evidence from Switzerland for instance suggests that workers with a labour market path characterised by full-time employment over a 20-year long period were less likely to experience mental health problems and depression than were those with a more unstable trajectory (Giudici and Morselli 2019). Having held temporary jobs between ages 25–45 has also been shown to be associated with subsequent depressive symptoms (Wahrendorf et al. 2019). Nevertheless, as with studying the link between static employment arrangements and mental health outcomes, trajectories over time should also be assessed in their complexity as encompassing a simultaneous exposure to multiple (and possibly interrelated) conditions of employment. To our knowledge, only a handful of studies have so far analysed the link between individuals’ employment trajectory over a longer exposure period and their mental health with an explicit multidimensional view grounded in employment quality and using a corresponding typological approach. One such study shows that low-quality labour market trajectories (characterised by time spent in multidimensionally defined precarious employment as well as unemployment) were linked to an increased risk of subsequent diagnosis of common mental disorders (depression, anxiety, and stress-related disorders), substance use disorders and suicide attempts in Sweden (Jonsson et al. 2021b). In a US study, an employment history characterised by poorer-quality employment and a gradual exit out of the labour force was also associated with an increased prevalence of moderate mental health problems among women (Eisenberg-Guyot et al. 2020). Such investigations show the merits of extending the scope of analysis from cross-sectional to longitudinal *and* multidimensional to better capture and analyse the nature of employment arrangements and potential mental health associations.

Our study aims to assess the link between the (multidimensional) quality of employment and subsequent mental ill-health using certified disabilities as a measure in Belgium. It focuses on individuals’ employment arrangement over time rather than exposure measured at one point in time, and does so drawing on administrative data from Belgium.

## Methods

### Data

Data for the analyses were derived from the Belgian Crossroads Bank for Social Security (CBSS) (CBSS—Datawarehouse Labour Market and Social Protection, n.d.). The CBSS data contain harmonised administrative information from various agencies tasked with delivering social security and social assistance in Belgium. The data spans from 2006 to 2016, with most variables reported on a quarterly basis though some (such as income) on an annual basis. An initial 10% random sample of the population of employees, jobseekers, early retirees, and those exempted from registering as jobseekers, aged 18–55 years known to Belgian social security institutions on 31 December 2005 (*n* = 366,624) was used. Ethics approval was granted to the project within which the investigation was carried out (reference number: ECHW_ 202).

Analytically, the time under study was divided into two periods: first an ‘assessment phase’ (2006–2009), during which individuals’ employment quality and trajectories were evaluated and, second, a follow-up phase (2010–2016), during which individuals’ risk of subsequent disability from mental ill-health was assessed.

### Study sample

To focus on the core working years throughout the 11 years under study, individuals aged 30–40 at baseline (on the last day of 2005) were eligible to be included in the analyses. Those who passed away or were assumed to have emigrated (indicated by missing register data) throughout the assessment phase were excluded. Individuals who had a known diagnosis of disability between 2006 and 2009 were also excluded to reduce the possibility of 'health selection', that is, that an existing health condition influences (non)employment opportunities (see Bartley et al. 2006). Those individuals categorised as ‘inactive/other’ during the assessment phase were also excluded from the analyses, allowing us to focus on the active labour force and to further lower the risk of health selection as we assume that it is in this category that individuals with emerging and under-diagnosed health problems are concentrated. The final sub-sample included 41,065 women and 45,667 men. A flow chart depicting the sub-sample selection can be found in the Supplementary Material (Fig. S1).

### Measures

#### Employment

Detailed information on individuals’ employment status was available on the last day of each quarter, based on which they could be categorised as (1) employees, (2) self-employed, (3) jobseekers/unemployed or (4) inactive (excluded; see above), and (5) other (excluded; see above). A detailed breakdown of the social security statuses within each employment status can be found in the Supplementary Material (Table S2). Employees with a main or complementary activity as self-employed were both categorised as self-employed. The quality of employment was then assessed using indicators representing four dimensions in each quarter: employment stability, multiple jobholding, working time, as well as income (described below). These dimensions are drawn from prior conceptualisations of employment quality and feature in scales of employment precariousness (Van Aerden et al. 2014, Koranyi et al. 2019, Jonsson et al. 2021a, Gevaert et al. 2021; Padrosa et al. 2021; Peckham et al. 2019; Vives et al. 2010). While there are more dimensions of employment quality signalled in this literature, data availability meant that only these four could be operationalised. For the first three dimensions, self-employed and jobseekers/unemployed were included as separate categories, whereas the waged-employed were sub-categorised as explained below. The income indicator was applied in a uniform way to the entire sample.

1. *Employment stability*. This indicator reflects whether an employee at any time within a given quarter:
  - held a *non-standard form of employment,* namely, temporary agency, seasonal, or any other specific forms of temporary work. Temporary agency and seasonal employment have a particular relative disadvantage in terms of mortality in Belgium (Balogh et al. 2021);
  - held a *subsidised form of employment*, such as working while retaining right to an income guarantee or working in an employment programme, as well as working while receiving an income replacement allowance for individuals due to health reasons (IVT). Albeit slightly different, working within the Belgian service voucher scheme of domestic work fell within the category of subsidised employment as well. Given previously highlighted concerns surrounding the quality of employment within the system, as well as the fact that this scheme is promoted as an entry point into the labour market, this choice can be justified (Mousaid et al. 2017);
  - showed indication of a *job change* (at least once) within the previous quarter. This was done as ‘regular’ fixed-term contracts between a worker and an employer could not be distinguished from open-ended contracts in this data set;
  - showed no indication of non-standard or subsidised employment, or any job change within the quarter (*stable*).
2. *Multiple jobholding*, while not incorporated in prior conceptualisations of employment quality due to it extending beyond the workplace, has been included as a dimension as its health implication has recently been investigated both in its own right and as part of employment precariousness (see Bouwhuis et al. 2019; Koranyi et al. 2019). This indicator reflects whether an employee on the last day of the quarter
  - *Held one job*, or
  - *Held two or more jobs* (multiple jobs).
3. *Working time* was approximated and expressed as a percentage of full-time hours equivalent (FTE) pertaining to the last day of the quarter, which we categorised as
  - *Less than 21% FTE*
  - *21–50% FTE*
  - *51–80% FTE*
  - *81–100% FTE*
  - *Over 100% FTE* (in case of multiple jobholding)
4. *Income* was expressed as quartiles on an annual basis, assuming an equal distribution within the year. It was approximated as follows. Information on income available in brackets of €5000 for income components separately (gross wage, self-employed income as well as various social security and social assistance payments derived from different institutions). First, we summed up the number of brackets for each year for all income components, then took the mid-point of the newly added-up bracket. For instance, an individual whose annual wage equalled 3 brackets of €5000 and who also received social security payments worth 2 brackets of €5000 had an approximated annual income of €22,500 (5 brackets in total, equalling 4 × 5000 + 2500). As a last step, we divided the entire sample’s distribution into quartiles (excluding individuals who passed away or were assumed to have emigrated during the assessment phase). The overview of the approximated ranges per income quartile and their distribution within the final sub-sample are shown in Table S1 in the supplementary material.

**Fig. 1:**
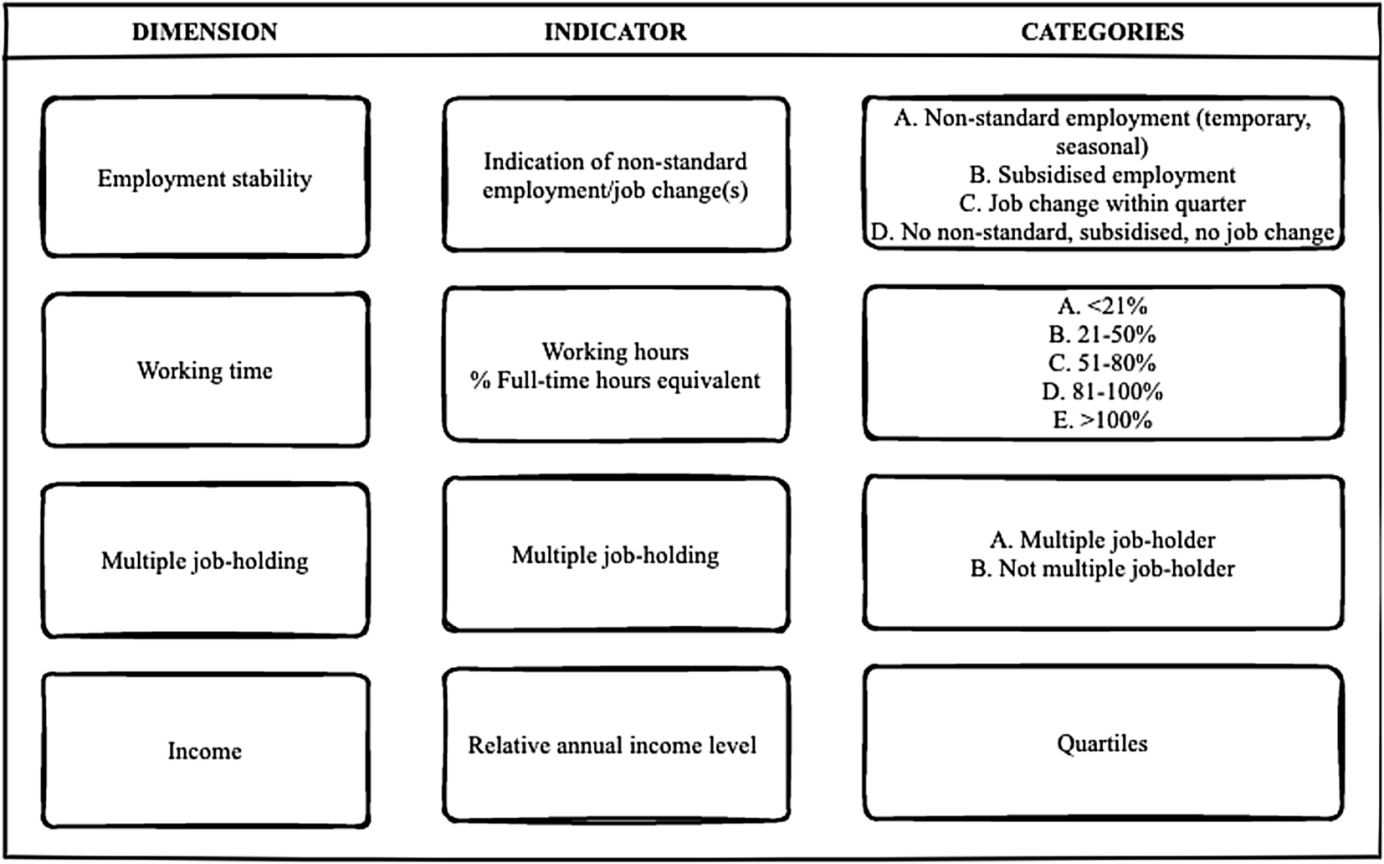
Overview of dimensions and corresponding indicators used to assess employment trajectories. Figure created with draw.io

An overview of the four dimensions and corresponding indicators assessed and incorporated in the subsequent clustering to construct types of trajectories (see below) is shown in Fig. 1. Further details on the construction of the indicators are supplied as Supplementary Material (Table S3).

#### Mental health

Diagnosis of a certified disability (certified by the Medical Council of Disability after an initial year of work incapacity on the advice of a medical doctor) (RIZIV 2021) was available on a quarterly basis. Diagnoses were grouped on a chapter basis using the International Classification of Diseases (ICD) 9th revision (from 2010 onward) and 10th revision (as of 2016). For disabilities related to mental health diagnoses, we used the following diagnoses: “Mental disorders” (ICD 9) and “Mental and behavioural disorders” (ICD 10). Note that individuals must have satisfied a certain waiting period and worked/were registered as jobseekers for a set number of days or hours to be eligible for work incapacity allowance, and that a separate scheme applies to employees, job-seekers and the self-employed (Vanroose 2019).

#### Covariates

When analysing the link between the type of employment trajectory over 2006–2009 and subsequent mental health-related disability, we adjusted for the following variables: age at baseline, household situation (whether the individual was living together with a partner at the end of 2009), and first nationality known to the registers (dichotomised as Belgian vs. non-Belgian). Individuals with no known nationality were assumed to not hold a (first) Belgian nationality.

### Methods

Employment trajectories were constructed using Multichannel Sequence Analysis, coupled with subsequent clustering. Multichannel Sequence Analysis extends traditional (Mono-channel) Sequence Analysis methods to allow for the simultaneous analysis of longitudinal sequences in separate—but often interrelated—areas (Gauthier et al. 2010). This method has recently been applied to the modelling of multidimensional employment trajectories in relation to health outcomes using US survey data (Eisenberg-Guyot et al. 2020). The four dimensions described above (employment stability, multiple jobholding, working time and income) were each considered a ‘channel’ within the Sequence Analysis, whereby the sequences of possible states within these four channels (e.g., working hours categories, income quartiles) were modelled. We implemented ‘longest common subsequence’ as the dissimilarity metric when computing the distances. This metric emphasises similarities in (and, therefore, attaches lower cost to) shared sequences of states (in our case, shared sequences of employment quality and status exposure), with substitution costs being set to 2 and insertion/deletion costs set to 1 (Gabadinho et al. 2011; Studer and Ritschard 2016).

Analyses were gender-stratified throughout the study (starting with the construction of trajectories), given the crucial way gender interacts with employment and mental health (see Valero et al. 2021).

After defining the sequence objects and computing the distances, Ward hierarchical clustering was applied to the distance matrix computed in the previous step to devise typologies of employment trajectories within the four dimensions of employment quality over the 16 quarters under study. Solutions with different numbers of clusters (from 2 to 12) were compared. Measures of partition quality (Rousseeuw 1987; Studer 2013), and knowledge, including prior typological studies, guided us when selecting the final cluster solutions. In short, applying Multichannel Sequence Analysis coupled with clustering allowed us to separate individuals within our sub-sample into groups (clusters), which, based on their sequences within the four employment quality features between 2006 and 2009 (and, of employment status, if not employed), were as similar as possible, while aiming to maximise dissimilarities between the trajectory clusters (Studer 2013). Deriving ideal types of jobs or employment arrangements—both cross-sectionally and longitudinally—with clustering methods is well-established in prior empirical research (Van Aerden et al. 2014, 2016, 2017; Jonsson et al. 2021a, b; Holman 2013; Eisenberg-Guyot et al. 2020; Peckham et al. 2019).

As a last step, cluster membership derived from the Sequence and cluster analyses (indicating the group to which an individual belonged based on their multidimensional trajectory between 2006 and 2009) was used to predict the hazard of experiencing disability due to a mental disorder during the follow-up phase (2010–2016). To this end, Cox proportional hazards regressions (Cox 1972) were fitted to calculate Hazard Ratios (HR) and corresponding 95% confidence intervals (CI). As the exact start date of the disability was not available, the mid-point of the quarter in which the first instance of a mental health-related diagnosis was indicated was taken as the ‘date of event’ (also called mid-point imputation, see Law and Brookmeyer 1992). Because, similarly, exact dates of death were not known, individuals who passed away during the follow-up phase were censored on the last day of their year of death. Individuals who (due to missing register data) were assumed to have emigrated between 2010 and 2016 were equally censored.

#### Sensitivity analyses

As the mid-point imputation we used to approximate the date of failure (the start of disability), albeit commonly used, is considered a less robust way of dealing with interval-censored data (see also Leffondré et al. 2013), a specific interval-censored Cox model was fitted as part of our sensitivity analyses. Further sensitivity analyses were conducted to probe into the robustness of our cause-specific findings by also looking at all-cause disability. In addition, Cox regressions were fitted to analyse the rate of mental health-related disability until the end of 2015 instead of the end of 2016. This analysis was to test whether a sudden rise in the number of mental and behavioural disorder diagnoses observed in the first quarter of 2016 might be biasing our results.

Data cleaning and survival analysis was performed using STATA versions 16.1 and 17.0, while sequence analysis was conducted in R (R Core Team 2021), with packages TraMiner, WeightedCluster and cluster (Maechler et al. 2021; Studer 2013; Gabadinho et al. 2011).

## Results

### Trajectory clusters

Multichannel Sequence Analyses revealed a number of trajectory clusters for women and for men. Initial solutions with 2 and 6 clusters were indicated to be better separated by some indices among women (see Fig. S2 in the Supplementary Material) yet they were unable to highlight ‘less prevalent’ and less ‘standard’ employment features which were dispersed amongst more standard and stable trajectories. Amongst women, 12 clusters were indicated to be relatively better separated by some partition quality measures, while a 10-cluster solution proved more parsimonious. By contrast, amongst men, the 12-cluster solution was most fitting given how well it outlined more vulnerable labour market groups and measures of partition quality. The clusters corresponding to the employment arrangements were labelled by the authors considering the distribution of different states (i.e., income and working hours categories) and mean times spent in each of those states in combination with theoretical knowledge. The labels highlight a characteristic that distinguishes a particular trajectory cluster from the others. Within the cluster we labelled as ‘Transitioning into self-employment’, for example, most individuals were considered employees working (near) full-time hours at the start of 2006 who nearly all moved into self-employment by the end of 2009 as shown in the state distribution graphs. These clusters are listed in Table 1.

**Table 1:** Overview of derived clusters, their labels, and main characteristics

Women		Men	
Name given to derived cluster	Characterised by…	Name given to derived cluster	Characterised by…
Standard	Single jobholding, constant decent income, working near full-time hours, little indication of non-standard/temporary employment or job change	Standard	Single jobholding, constant decent income, working near full-time hours, little indication of non-standard/temporary employment or job change
Modest income standard	Single jobholding, lower relative income (mostly lowest and 2nd quartile), working near full-time hours with some probability of part-time, little indication of non-standard/temporary employment or job change	Income mobility	Divergent and potentially changing relative income over the 16 quarters of assessment period
Favourable PT	Single jobholding, low probability of lowest income quartile, mostly 51–80% FTE working hours, little indication of non-standard/temporary employment or job change	Multiple jobholder	Increased probability of multiple jobholding and working above 100% FTE hours
High earner	Single jobholding, highest relative income, mostly near full-time hours, little indication of non-standard/temporary employment or job change	High earner	Single jobholding, highest relative income, near full-time hours, little indication of non-standard/temporary employment or job change
Unstable	Increased probability of unemployment/subsidised or non-standard forms of employment over the 16 quarters of assessment period, lower relative income, varied working hours	Slowly converging	Income levels that do not necessarily change in line with others’ rate of income rise
Unfavourable PT	Single jobholding, high probability of lowest income quartile, mostly 51–80% FTE working hours, little indication of non-standard/temporary employment or job change	Transitioning into SE	Likely transition from paid employment to self-employment by the end of the assessment period
Low hours	Single jobholding, high probability of lowest income quartile, mostly 21–50% FTE working hours, little indication of non-standard/temporary employment or job change	Modest income	Single jobholding, 2nd income quartile, near-full time FTE hours, little indication of non-standard/temporary employment or job change
Multiple jobholder	Increased probability of multiple jobholding and working above 100% FTE hours	Fluctuating high-income	Increased probability of moving from and to 3rd and highest income quartile
Transitioning into SE	Likely transition from paid employment to self-employment by the end of the assessment period	Weaker attachment	Increased probability of part-time working hours
Unemployed	Near-constant unemployment over the 16 quarters of assessment period	Self-employed	Quick transition to and/or near-constant self-employment over the 16 quarters of assessment period
		Unstable	Increased probability of unemployment and subsidised or non-standard forms of employment over the 16 quarters of assessment period, lower relative income, varied working hours
		Unemployed	Near-constant unemployment over the 16 quarters of assessment period

Some trajectory types were outlined among both men and women, such as the ‘Standard’ (essentially constant full-time employment, and decent income), ‘High earner’ (analogous to ‘Standard’ but with the highest relative income), ‘Multiple jobholder’ (high probability of multiple jobholding) and ‘Unemployed’ trajectories. Others were gender specific. As might be expected, more groups characterised by part-time employment were found among women. However, they differed based on the relative annual income (‘Unfavourable part-time’, ‘Favourable part-time’), as well as working hours (‘Low hours’). The ‘Unstable’ trajectory, although given the same name, showed slightly different characteristics by gender. Amongst both men and women, an increased probability of experiencing unemployment is evident. In addition, amongst women, it involves a significant proportion of workers undertaking subsidised employment (mainly through the service voucher scheme) at one point between 2006 and 2009. A trajectory characterised by a gradual entry into self-employment was found among both men and women (‘Transitioning into self-employment’), whereas some men made this career switch closer to the start of the study (‘Self-employed’). Some variations in terms of the level and dynamics of annual relative income were also highlighted in the different typologies of 4-year trajectories (‘Modest income standard’ among women, ‘Income mobility’, ‘Slowly converging’, and ‘Modest income’ among men).

The derived clusters are presented in Tables 2 and 3 as state distribution graphs along with their given labels, and mean times spent in each state are also shown in the Supplementary Material (Tables S5 and S6). These graphs plot the distribution of different states (of income, multiple jobholding, number of working hours and stability) within the particular cluster (on the *y* axis) over time (*x* axis). The vertical bars correspond to the 16 quarters over the assessment period. Note, therefore, that such graphs do not plot individual trajectories horizontally.

**Table 2:**
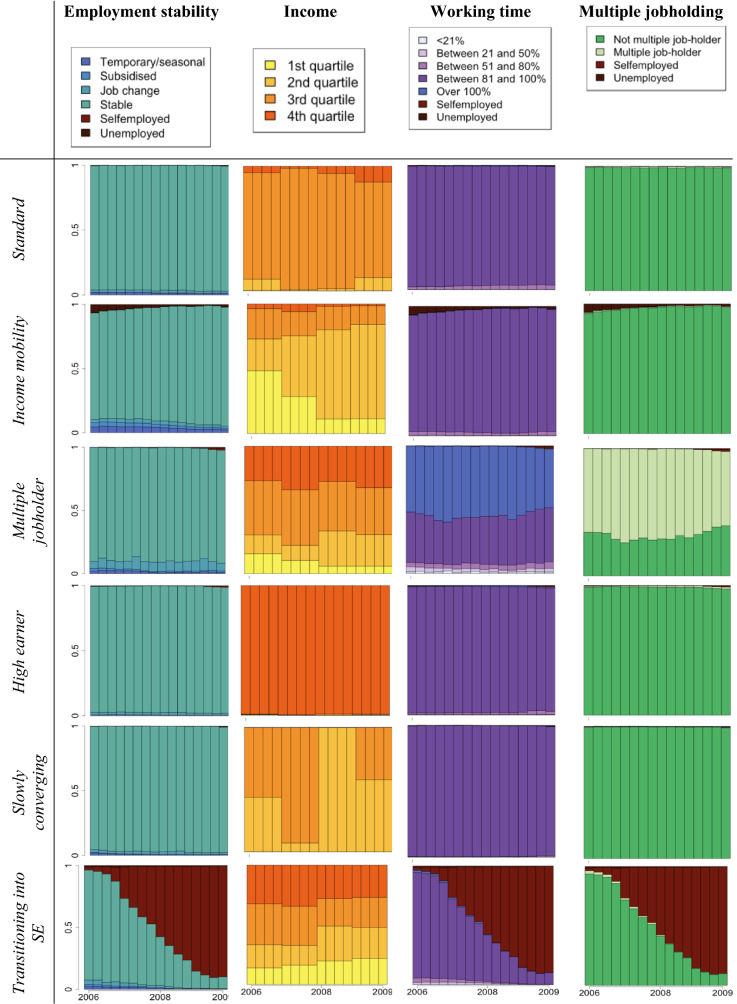

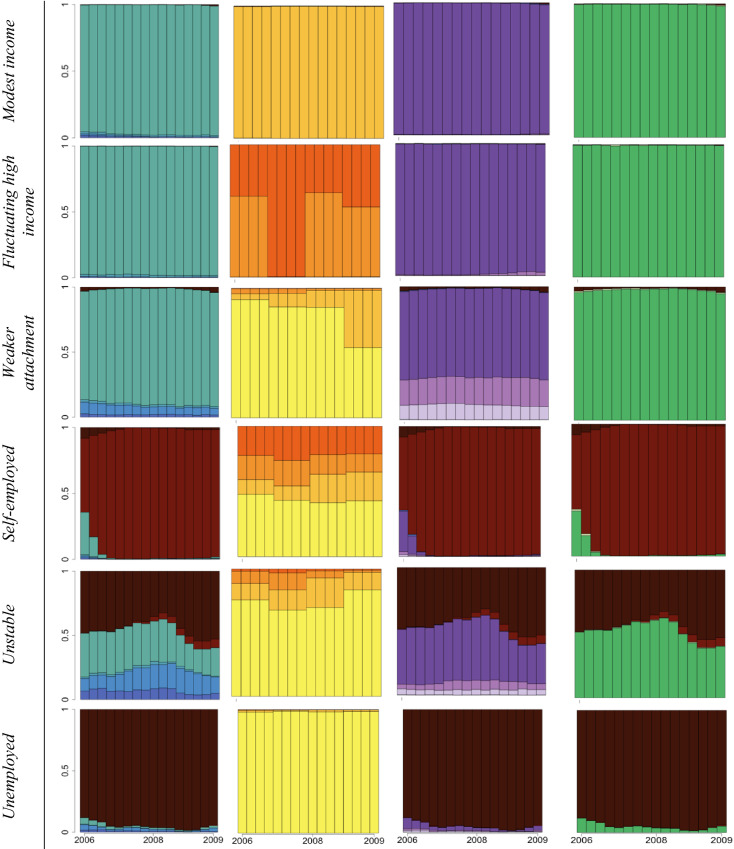
State distribution graphs of employment trajectory characteristics 2006–2009 by clusters among men. Source: CBSS

*SE* self-employment

**Table 3:**
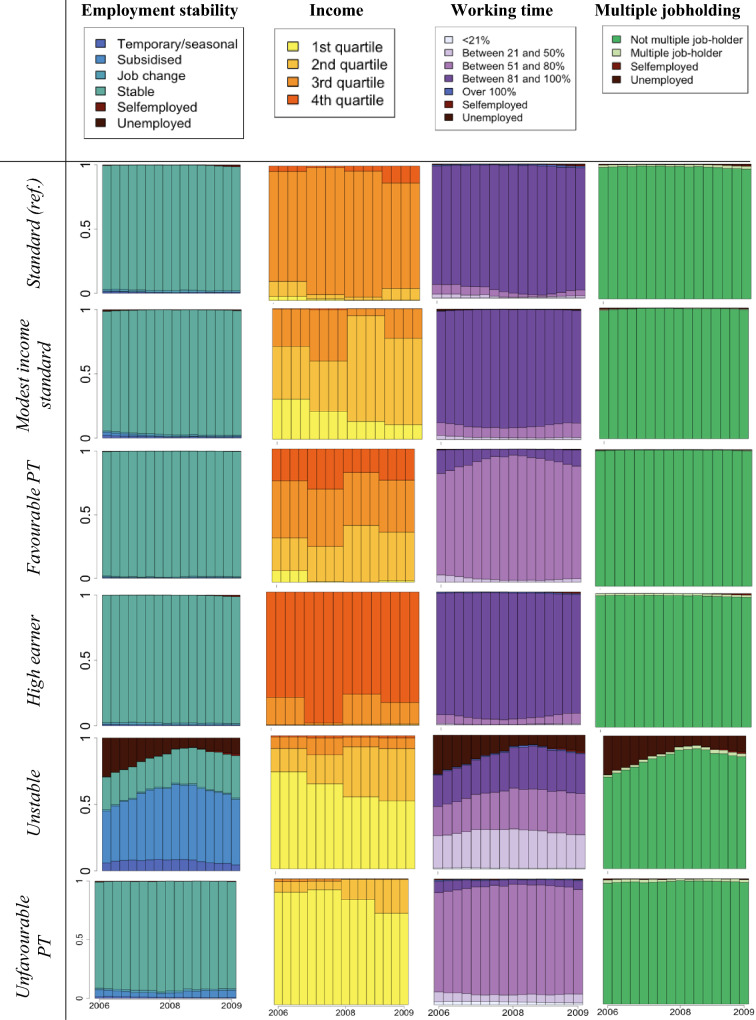

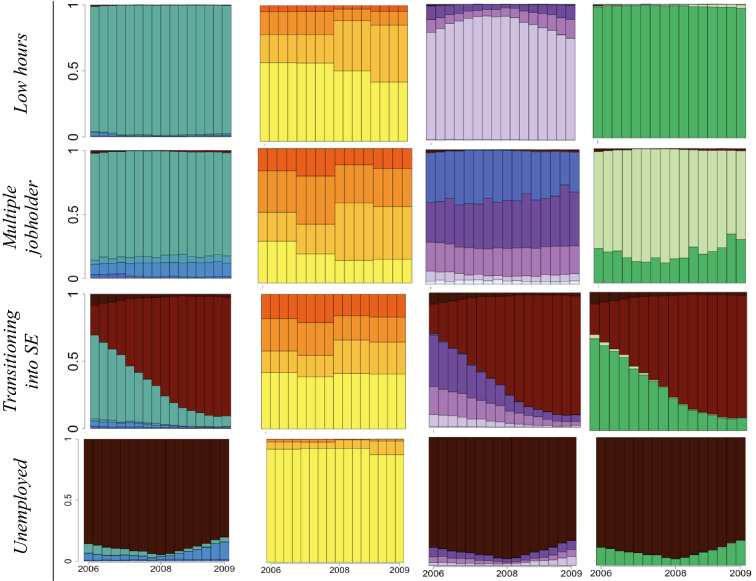
State distribution graphs of employment trajectory characteristics 2006–2009 by clusters among women. *Source*: CBSS

*PT *part-time, *SE *self-employment

Descriptive statistics (shown in Tables S3 and S4 in the Supplementary material) show a higher proportion of individuals with non-Belgian first nationality amongst those with an ‘Unstable’ trajectory or in near-constant unemployment between 2006 and 2009 than among those with a ‘Standard’ or ‘High earner’ labour market path.

### Associations between trajectory type and mental health outcome

Survival analyses revealed a clear cause-specific disability gradient among both men and women according to trajectory type, as shown in Tables 4 and 5 for men and women, respectively. The trajectory type labelled as ‘Standard’ was used as a reference category among both men and women. Proportional hazards were assessed, and no violation of this assumption was detected.

**Table 4:** Associations between type of employment trajectory 2006–2009 and disability from mental disorder 2010–2016 among men. *Source*: CBSS

	*n*	*n* of cases of mental health-related disability 2010–2016	Unadjusted regression estimates	Adjusted regression estimates
Standard (ref.)	8292	59	1.00	1.00
Income mobility	6211	124	2.84***	2.77***
			(2.08, 3.87)	(2.03, 3.78)
Multiple jobholder	732	8	1.55	1.51
			(0.74, 3.24)	(0.72, 3.16)
High earner	10,558	57	0.76	0.78
			(0.53, 1.09)	(0.54, 1.12)
Slowly converging	5860	69	1.66**	1.63**
			(1.17, 2.35)	(1.15, 2.31)
Transitioning into SE	1891	15	1.12	1.17
			(0.63, 1.97)	(0.66, 2.05)
Modest income	3252	41	1.78**	1.68*
			(1.19, 2.65)	(1.12, 2.50)
Fluctuating high-income	3793	30	1.11	1.12
			(0.72, 1.73)	(0.72, 1.74)
Weaker attachment	2337	55	3.35***	3.05***
			(2.32, 4.84)	(2.11, 4.42)
Self-employed	842	9	1.51	1.49
			(0.75, 3.04)	(0.74, 3.02)
Unstable	949	51	7.90***	7.01***
			(5.43, 11.49)	(4.79, 10.27)
Unemployed	950	68	10.70***	8.64***
			(7.55, 15.17)	(6.03, 12.40)
Belgian first nationality (ref: non-Belgian)				1.00
				(0.82, 1.22)
Partner in household (ref: no partner in household)				0.61***
				(0.51, 0.72)
Age (continuous)				1.03*
				(1.00, 1.05)

Hazard Ratios (and 95% confidence intervals) from Cox proportional hazards regressions

Exponentiated coefficients; 95% confidence intervals in brackets

*SE *self-employment

^*^p < 0.05, **p < 0.01, ***p < 0.001

**Table 5:** Associations between type of employment trajectory 2006–2009 and disability from mental disorder 2010–2016 among women. *Source*: CBSS

	*n*	*n* of cases of mental health-related disability 2010–2016	Unadjusted regression estimates	Adjusted regression estimates
Standard (ref.)	5938	97	1.00	1.00
Modest income standard	8476	201	1.46**	1.43**
			(1.15, 1.86)	(1.12, 1.82)
Favourable PT	5550	112	1.24	1.42*
			(0.94, 1.62)	(1.08, 1.87)
High earner	8218	124	0.92	0.96
			(0.71, 1.21)	(0.74, 1.26)
Unstable	3180	171	3.37***	2.94***
			(2.63, 4.32)	(2.29, 3.78)
Unfavourable PT	3089	94	1.88***	2.05***
			(1.41, 2.49)	(1.54, 2.73)
Low hours	3074	77	1.54**	1.79***
			(1.14, 2.08)	(1.32, 2.42)
Multiple jobholder	809	15	1.14	1.09
			(0.66, 1.96)	(0.63, 1.87)
Transitioning into SE	1513	39	1.59*	1.60*
			(1.09, 2.30)	(1.10, 2.32)
Unemployed	1218	125	6.64***	5.20***
			(5.09, 8.65)	(3.96, 6.81)
Belgian first nationality (ref: non-Belgian)				0.78**
				(0.67, 0.91)
Partner in household (ref: no partner in household)				0.53***
				(0.47, 0.60)
Age (continuous)				0.99
				(0.98, 1.01)

Hazard Ratios (and 95% confidence intervals) from Cox proportional hazards regressions

Exponentiated coefficients; 95% confidence intervals in brackets

*PT *part-time, *SE *self-employment

^*^p < 0.05, **p < 0.01, ***p < 0.001

Amongst men, two particular groups revealed higher levels of risks of disability from mental ill-health after adjusting for first nationality, age, and partnership situation. These two groups are, first, the ‘Unstable’ group (Hazard Ratio (HR): 7.01, 95% confidence interval (CI): 4.79, 10.27) due to a heightened probability of unemployment spells and characterised by lower relative income and potential non-standard employment and, second, the ‘Unemployed’ labour market trajectory cluster (HR: 8.64, 95% CI 6.03, 12.40). Those with a trajectory characterised by ‘Income mobility’ (HR: 2.77, 95% CI 2.03, 3.78), ‘Modest income’ (HR: 1.68, 95% CI 1.12, 2.50), ‘Slowly converging’ income (HR: 1.63, 95% CI 1.15, 2.31), and ‘Weaker attachment’ (HR: 3.05, 95% CI 2.11, 4.42) also demonstrated a mental health disadvantage in terms of disability diagnosis.

Apart from those in high earner and multiple jobholder trajectories, all other clusters among women had a heightened risk of having registered disability due to mental ill-health between 2010 and 2016 compared to the ‘Standard’ trajectory after adjustment. As among men, two trajectory types amongst women had a particularly increased risk. The ‘Unstable’ trajectory, characterised by a high prevalence of subsidised work (in particular within the service voucher scheme), potentially interspersed with spells of unemployment, had a threefold higher hazard of experiencing a disability from mental disorders after adjustment (HR: 2.94, 95% CI 2.29, 3.78) compared to the ‘Standard’ trajectory, whereas the (near-)constant unemployed had a fivefold higher hazard (HR: 5.20, 95% CI 3.96, 6.81).

### Sensitivity analyses

The results of sensitivity analyses are shown as Supplementary material. Overall, these analyses confirm core patterns found in our main analyses. Fitting Cox models considering interval censoring yielded near-identical adjusted estimates as our original analyses using mid-point imputation (Tables S7 and S8). Similar patterns for all-cause disability were found as in our cause-specific analyses focusing on mental disorders (Tables S9 and S10). However, high earners had a lower risk of all-cause disability than those in a standard trajectory, and men with an ‘Unstable’ trajectory and unemployed males had lower hazard ratios for all causes than for mental disorders. Being a female multiple jobholder between 2006 and 2009 was a predictor for all-cause disability between 2010 and 2016, while this was not the case for mental disorders. Further robustness checks—as shown in Tables S11 and S12—reveal that the increase in new mental disorder diagnoses at the beginning of 2016 likely did not affect our original estimates. In fact, higher risk estimates were found for men in “Unstable” and “Unemployed” trajectories when using the end of 2015 as a cutoff, although this reflects a lower number of total cases.

## Conclusions and discussion

Longitudinal studies on employment quality and mental health are needed to further explore the association established in the literature. Our study explored various employment trajectories over 16 quarters between 2006 and 2009 in Belgium and investigated the links between the type of an individual’s trajectory and their hazard of a subsequent mental health-related disability. By considering multiple aspects of employment and income over time, we have been able to separate ideal types of secure and stable, as well as vulnerable and unstable employment trajectories, which cross-sectional and mono-dimensional investigations are not able to uncover. In addition, incorporating jobseekers/the unemployed and the self-employed has enabled us to provide a more comprehensive understanding of the relationship between employment trajectory, employment quality and mental health amongst the Belgian labour force.

We have shown an apparent gradient of mental ill-health leading to a disability across the Belgian labour force aged 30–40 at baseline. The ‘Unemployed’ and those with an ‘Unstable’ trajectory (a higher proportion of time spent in subsidised and non-standard employment, with potential spells of unemployment and lower relative income) demonstrated a significant subsequent mental health disadvantage. While we deliberately avoided using the term ‘precarious employment’ for trajectory types to emphasise differences in our approximation of employment quality compared to previous investigations, the health inequality found adds to the body of evidence on the adverse implications of employment arrangements that are often labelled ‘precarious’ (Peckham et al. 2019; Vives et al. 2011, Van Aerden et al. 2016, 2017). Our work is the latest in a small number of studies which have examined moderate mental illness (Eisenberg-Guyot et al. 2020) as well as common mental disorders, substance use and suicide attempts (Jonsson et al. 2021b) in relation to multi-dimensional trajectories of employment quality. However, our study is the first to investigate this link in the Belgian context, looking at diagnosed cause-specific disability and employment information derived from administrative data incorporating mental health-related registered disability.

One of the strengths of this study lies in its gender stratified perspective. Our approach has enabled us to show that vulnerable trajectories can be characterised differently depending on gender, making a case for typological approaches to be completely gender stratified (and gender sensitive). Women transitioning into self-employment during the 4 years under study exhibited increased risk of a mental health-related disability, whereas their male counterparts in, or transitioning into, self-employment, did not. It might be that more women than men in this group might be moving into a less advantageous form of self-employment in terms of employment quality and mental health, such as ‘dependent’ or ‘insecure’ self-employment, which might explain these differences (see Gevaert et al. 2021). Differences in terms of eligibility and replacement income between the wage-employed and self-employed, however, might also influence differences in disability diagnoses or take-up of replacement income between the two groups. Divergent leading causes of disability have indeed been observed between the self-employed and wage-employed in Belgium (RIZIV 2018).

Those exposed to near-constant unemployment and a more unstable trajectory exhibited elevated rates of subsequent mental health-related disability compared to those in a more standard trajectory. This difference was also more pronounced for men compared to their female counterparts in similar trajectories. A possible explanation lies in the heterogeneity of the clusters (see also below): while among men, the ‘Unstable’ trajectory included a more selective group of individuals with heightened employment insecurity, the ‘Unstable’ group among women also comprised individuals undertaking continuous work within the service voucher scheme without any unemployment spells. Men without stable, secure employment might also be more severely affected than their female counterparts due to the likelihood that they are the primary earners within their household (see also Van Lancker 2012). It is also important to point out the confounding and possible selection effects that arose from our inability to adjust for socio-economic and health-related factors in our analyses. This limitation might also have implications for the gender-specific patterns we found for the risk of developing a serious mental disorder, as gendered differences in the socio-economic or health background of workers in various trajectories might as well be at play.

Multiple jobholders did not show an increased risk of being diagnosed with mental disorder-related disability compared to the standard trajectories. Some suspected reasons behind the null associations found for multiple jobholding and long-term sickness might also be at play in our study: this includes multiple jobholders being healthier to begin with, and the group comprising various kinds of workers (Bouwhuis et al. 2017).

Administrative data such as that from which we draw our sample has the potential of contributing to evidence on labour market trajectories and health. It can allow for the construction of employment histories including detailed movements with less concern arising regarding recall bias and allows researchers to determine the timing and length of exposure to a certain employment status more precisely (see Bodin et al. 2020). Moreover, diagnostic measures of mental health are an important complement to evidence using self-reported indicators.

Our study, however, also reveals the limits of using current administrative data. Our employment trajectories had to be composed based on a more limited number of indicators of employment quality than those usually available in surveys. Dimensions including the vulnerability of the employee or information on the actual hours worked (including overtime) or how overall rights are exercised (Padrosa et al. 2021; Van Aerden et al. 2014; Vives et al. 2010) are not currently available due to data limitations and so could not be incorporated in our analyses. These omissions potentially limit understanding of the most precarious forms of employment. Individual-level income on an annual basis also had to be approximated from the data which will entail a certain level of imprecision. Similarly, we only had available a narrow range of background characteristics. Adjusting for known confounders such as intrinsic work characteristics or highest educational attainment would have likely attenuated the extent of associations we have found (Van Aerden et al. 2017; Balogh et al. 2021).

In our analysis, the estimates likely also capture broader socioeconomic and class differences and processes (Muntaner et al. 2004; Prins et al. 2021) that are encapsulated in less standard employment trajectories, factors that we were not able to adjust for. In addition, we cannot rule out ‘health selection effects’—i.e., the possibility that some individuals had an underlying (mental) health condition that limited their employment opportunities (see Bartley et al. 2006). Indeed, recent evidence suggests that poor mental health might lead individuals to leave permanent employment in favour of a temporary job (Dawson et al. 2015). We were able to partially mitigate such selection effects by excluding the inactive and persons with a disability diagnosis during our assessment phase. Nevertheless, this limitation is further underscored by the fact that the diagnostic measure on which we relied to assess mental health captured serious cases only, that is, certified disabilities in Belgium are generally preceded by a year of primary incapacity (RIZIV 2021). Findings should be interpreted bearing in mind that subsidised forms of employment can also mean that an individual receives an income guarantee for a reason which contributes to health selection. Adjusting for self-reported (mental) health status prior to or during our assessment phase would have been a more effective way to deal with selection effects but our data did not allow us to do so. Therefore, mental disorders that did not lead to a disability or remained undiagnosed in the assessment phase could not be captured and adjusted for.

Hypothesised health pathways between precarious employment and health outcomes might also apply in our case and mediate the influence of longer term employment situation on mental health. Pathways can include immediate psychosocial mechanisms (Vanroelen et al. 2021). Low (or volatile) income can also affect workers’ and their household members’ material conditions which deprivation in turn can lead to adverse (mental) health (Benach et al. 2014; Vanroelen et al. 2021). We leave the task of disentangling the detailed causal pathways between employment quality and mental health to future studies. In sum, however, health selection and socio-economic confounding might partly explain the fact that a large number of clusters were linked to an elevated risk of mental disorder compared to the ‘standard’ reference group.

One further limitation that needs to be borne in mind relates to the—potentially volatile—separation of clusters. As with a prior study applying Multichannel Sequence Analysis to the modelling of employment trajectories (Eisenberg-Guyot et al. 2020), the (weighted) Average Silhouette Width (a measure of partition quality) was relatively low for our cluster solutions (below 0.5), indicating potentially volatile cluster structures (Rousseeuw 1987; Studer 2013). Decomposition by group shows that it is mainly due to the ‘Unstable’ trajectories showing low within-group homogeneity, likely because different kinds of more non-standard trajectories are grouped together in those clusters (data not shown). While heterogeneity within some groups does limit the conclusions one can draw regarding health associations, we do believe our results highlight some preliminary work- and health-related inequalities that need to be investigated further.

An important analytical contribution that our study makes pertains to the conceptualisation and measurement of low quality and precarious employment arrangements over time. Our study suggests that ‘low-quality/precarious employment’ might be hard to study separately from unemployment given movements across the labour market. Future research should seek to maintain a longer exposure period of employment arrangements and attendant employment quality. Precarity over time could manifest differently from precarity seen at one point in time: precariousness is not merely a repeated exposure to a certain employment arrangement but involve dynamics of employment progression or stagnation or even decline as well as unpredictability and insecurity (Fuller 2009).

In addition, our study adopted an individual-centred approach and was not able to assess aspects such as household-level income due to lack of information. Future research might also seek to include the household as an additional unit of analysis when investigating work-related mental health. This inclusion would be useful as adverse employment arrangements can have adverse spill-over consequences for individuals’ lives that extend beyond the individual and beyond the workplace (Della Porta et al. 2015; Matilla-Santander et al.  2022; Quinlan and Bohle 2015).

Despite its limitations, this paper has considerable strengths. We have demonstrated that mental disorder-disabilities are most likely not randomly distributed across the labour force in Belgium. Those individuals who are exposed to a considerable length of unemployment between 2006 and 2009 and individuals with a trajectory involving a higher probability of subsidised or non-standard employment and spells of unemployment demonstrated the highest subsequent mental health disadvantage as indicated by a registered disability. Men with a lower relative individual income over the period of 4 years and some labour market trajectories characterised by mainly part-time employment among women are also associated with an increased risk of mental health-related disability. Future research should examine the mechanisms and the extent to which these associations can be ascribed to exposure to certain (un)employment arrangements or whether selection effects are the primary movers of these associations and explore other avenues in trajectory clustering. We also showed the possibilities of using administrative data in assessing work-related mental health inequalities. Such data can capture some groups that might be harder to reach via surveys. Finally, given the increasing move toward longitudinal analyses on employment and health, this paper presents an important step in thinking on how to best conceptualise and measure precarious employment trajectories.

## Supplementary Information

Below is the link to the electronic supplementary material.

Supplementary file 1 (DOCX 274 kb)

## Data Availability

The data that support the findings of this study are available from the Crossroads Bank for Social Security, but restrictions apply to the availability of these data, and so are not publicly available.
