# Supplementary material for: Employment trajectories and mental health-related disability in Belgium
Source: Int Arch Occup Environ Health. 2022 Oct 10;96(2):285–302. doi: 10.1007/s00420-022-01923-y (PMC9905181; doi:10.1007/s00420-022-01923-y)
Supplement: Supplementary file 1 — (DOCX 274 kb) [file 420_2022_1923_MOESM1_ESM.docx]

Supplementary material for manuscript “*Employment trajectories and mental health-related disability in Belgium”*

Table of Contents

Figure S1 - Flow chart of sub-sample 2

Figure S2 - Overview of partition quality indicators per cluster solutions 3

Table S1 - Overview of personal annual income per year 4

Table S2 – Overview of employment status categories in the register 6

Table S3 - Construction of employment quality indicators 8

Table S3 – Employment trajectory type and background characteristics among men 9

Table S4 – Employment trajectory type and background characteristics among women 10

Table S5 - Average time (expressed in quarters) spent in each state by employment trajectory cluster among men between 2006 and 2009 (total=16). 11

Table S6 - Average time spent (expressed in quarters) in each state by employment trajectory cluster among women between 2006 and 2009 (total=16). 12

Table S7 - Associations between type of employment trajectory 2006-2009 and disability from mental health disorder 2010-2016 among men. Hazard ratios (and 95% confidence intervals) from Cox proportional hazards regressions using interval censoring. Sensitivity analysis 13

Table S8- Associations between type of employment trajectory 2006-2009 and disability from mental health disorder 2010-2016 among women. Hazard ratios (and 95% confidence intervals) from Cox proportional hazards regressions using interval censoring. Sensitivity analysis 14

Table S9 - Associations between employment trajectories and all-cause disability 2010-2016 among men. Sensitivity analyses 15

Table S10 - Associations between employment trajectories 2006-2009 and all-cause disability 2010-2016 among women. Sensitivity analyses 16

Table S11 - Associations between type of employment trajectory 2006-2009 and disability from mental health disorder 2010-2015 among men. Hazard ratios (and 95% confidence intervals) from Cox proportional hazards regressions. Sensitivity analysis 17

Table S12 - Associations between type of employment trajectory 2006-2009 and disability from mental health disorder 2010-2015 among women. Hazard ratios (and 95% confidence intervals) from Cox proportional hazards regressions. Sensitivity analysis 18


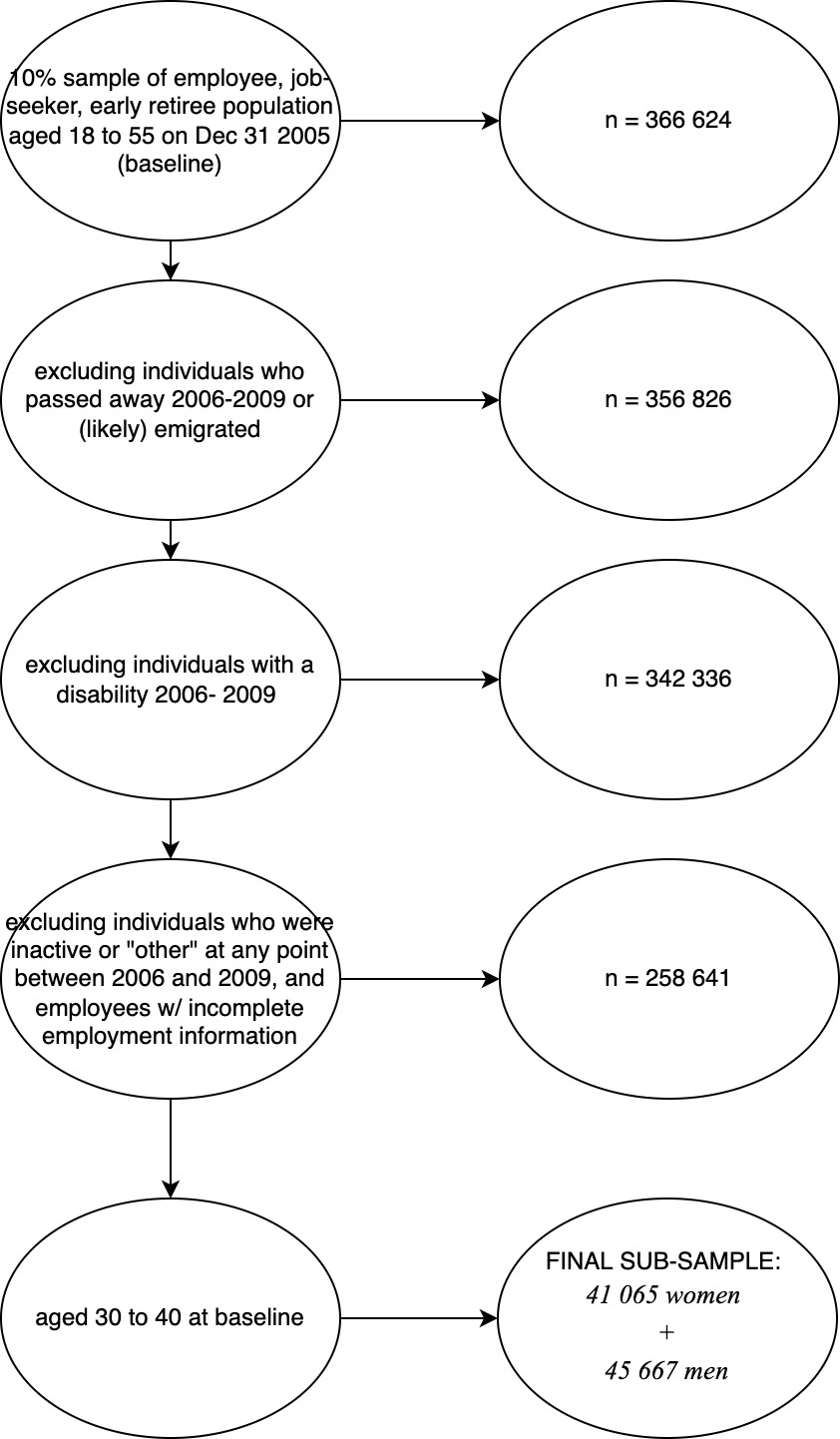


# Figure S1 - Flow chart of sub-sample


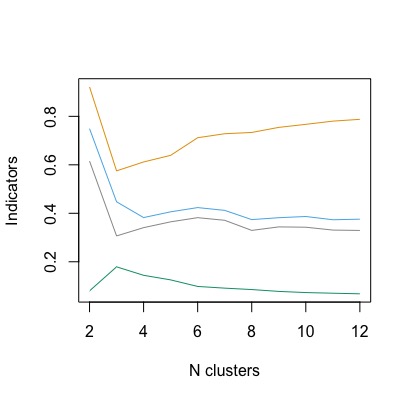

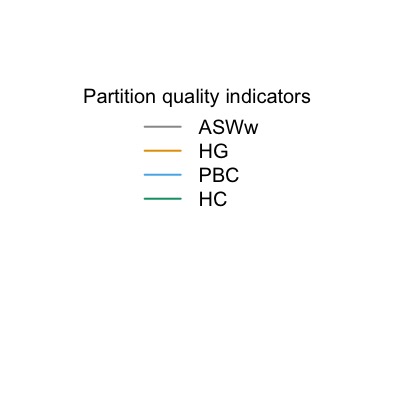

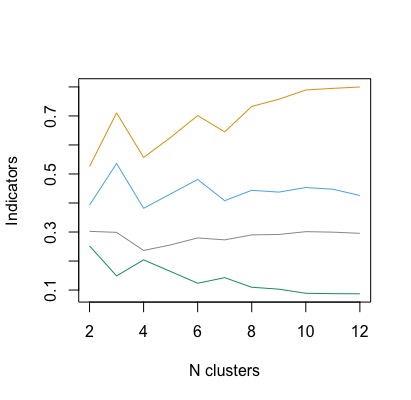


# Figure S2 - Overview of partition quality indicators per cluster solutions

(women on the right, men on the left). ASWw: Weighted Average Silhouette Width, HG: Hubert’s Gamma, PBC: Point Biserial Correlation, HC: Hubert’s C.

Note: Higher values indicate better partition quality, except for HC. See Studer, 2013. Produced with TraMiner package in R

| **MEN** | | | | | | | | | | | | |
| --- | --- | --- | --- | --- | --- | --- | --- | --- | --- | --- | --- | --- |
|  | **2006** | | | **2007** | | | **2008** | | | **2009** | | |
|  | N | Range min. (€) | Range max. (€) | N | Range min. (€) | Range max. (€) | N | Range min. (€) | Range max. (€) | N | Range min. (€) | Range max. (€) |
| **Quartile 1** | 7666 | 1 | 25000 | 6119 | 1 | 25000 | 5066 | 1 | 25000 | 4550 | 1 | 25000 |
| **Quartile 2** | 8895 | 25001 | 30000 | 7549 | 25001 | 30000 | 15009 | 25001 | 35000 | 14085 | 25001 | 35000 |
| **Quartile 3** | 15495 | 30001 | 40000 | 16044 | 30001 | 40000 | 12248 | 35001 | 45000 | 12732 | 35001 | 45000 |
| **Quartile 4** | 13611 | 40001 | maximum | 15955 | 40001 | maximum | 13344 | 45001 | maximum | 14300 | 45001 | maximum |
| **WOMEN** | | | | | | | | | | | | |
|  | **2006** | | | **2007** | | | **2008** | | | **2009** | | |
|  | N | Range min. (€) | Range max. (€) | N | Range min. (€) | Range max. (€) | N | Range min. (€) | Range max. (€) | N | Range min. (€) | Range max. (€) |
| **Quartile 1** | 12131 | 1 | 25000 | 10423 | 1 | 25000 | 8982 | 1 | 25000 | 8083 | 1 | 25000 |
| **Quartile 2** | 7432 | 25001 | 30000 | 6971 | 25001 | 30000 | 13036 | 25001 | 35000 | 12389 | 25001 | 35000 |
| **Quartile 3** | 12831 | 30001 | 40000 | 13099 | 30001 | 40000 | 11086 | 35001 | 45000 | 11155 | 35001 | 45000 |
| **Quartile 4** | 8671 | 40001 | maximum | 10572 | 40001 | maximum | 7961 | 45001 | maximum | 9438 | 45001 | maximum |

# Table S1 - Overview of personal annual income per year

| **Employment status in the register** | **Categorised as** |
| --- | --- |
| 1. Working |  |
| 1.1. Working as an employee | ***Employee*** |
| 1.1.1. Working in one job in salaried employment |  |
| 1.1.2. Working in multiple paid jobs |  |
| 1.2. Working as a self-employed person | ***Self-employed*** |
| 1.2.1. Self-employed in main occupation |  |
| 1.2.2. Self-employed in secondary occupation |  |
| 1.2.3. Working as a self-employed person after retirement (age) |  |
| 1.3. Working as a helper for an employer with self-employed status |  |
| 1.3.1. Working as a helper in main profession |  |
| 1.3.2. Working as a sideline helper |  |
| 1.3.3. Working as a helper after retirement (age) |  |
| 1.4. Working as an employee and as a self-employed person/helper |  |
| 1.4.1. Main job is in paid employment |  |
| 1.4.2. Main job is performed as a self-employed person in main occupation with an additional job as an employee |  |
| 1.4.3. Main job is performed as a helper in main occupation with an additional job in paid employment |  |
| 2. Job-seeker |  |
| 2.1. Job-seeker after full-time employment, with unemployment benefits | ***Unemployed*** |
| 2.2. Job-seeker after a voluntary part-time job, with unemployment benefits |  |
| 2.3. Job-seeker after studies, with waiting allowance or bridging allowance (before 2012) |  |
| 2.4. Job-seeker with guidance benefit |  |
| 3. Not professionally active (inactive) |  |
| 3.1. Full Career Break / Full Time Credit | ***Inactive - excluded 2006-2009*** |
| 3.2. Exemption from registration as a jobseeker |  |
| 3.3. "Leefloon"/financial aid |  |
| 3.3.1 "Leefloon" |  |
| 3.3.2 Financial aid |  |
| 3.4. Retired without work |  |
| 3.5. Fully retired |  |
| 3.5.1 Full early retirement (before 2012) |  |
| 3.5.2 Availability prior to retirement |  |
| 3.6. Children entitled to child benefit |  |
| 3.7. Incapacitated for work |  |
| 3.7.1 Disability known to the health insurance companies |  |
| 3.7.2 Incapacitated for work due to disability |  |
| 3.7.3 Incapacitated for work due to an occupational disease |  |
| 3.7.4 Incapacitated for work due to an accident at work |  |
| 3.8 Person with a disability allowance |  |
| 4. Other | ***Other - excluded 2006-2009*** |

# Table S2 – Overview of employment status categories in the register (CBSS)

|  | **Dimension** | **Indicator derived** | **Construction method - description** | **Variables used (and supplying institution)** |
| --- | --- | --- | --- | --- |
| **Employees** | **Employment stability** | Temporary agency work | approximated as a combination of sector number (temp agency are a separate sector in Belgium) and type of contract ("special") for an employment performance in a given quarter | nr_cp, t_prest (RSZ) |
|  |  | Temporary work (other) | Indication of some form of temporary work for employees covered by the Social Security Institution for municipal employees | Status (RSZ PPO) |
|  |  | Seasonal work | indication of seasonal work in a given quarter | not_sai (RSZ) |
|  |  | Subsidised/employment promotion program | indication of participation in any subsidised/employment promotion programs, or working while receiving a guaranteed income | activation_ONEm, ALE, pod_mi_travailleur, fodsz_travailleur, alloc_garantie_rev (KSZ), EmploymentPromotion (RSZ PPO) |
|  |  | Service voucher scheme work | indication of working in the service voucher scheme | Proximity_jobs (RSZ) |
|  |  | Job change | indication of discrepancy between number of employment performances reported in a given quarter and number of jobs held on the last day of quarter | jobnummer and corresponding variables pertaining to the employment performance (RSZ, RSZ PPO), jobs_tot (KSZ) |
|  | **Multiple job-holding** | Holding more than one job | Holding more than one job on the last day of a given quarter. Note that temporary agency workers were coded as single job-holders | jobs_tot (KSZ) |
|  | **Working time** | Working hours | Approximation of working hours as % of FTE hours (compared to an 'average worker') worked on the last day of a quarter | cum_pct, tauxpt, apltauxpt, t_prest (KSZ, RSZ and RSZ PPO) |
|  | **Income** | Relative annual income level | Approximation of annual income through brackets of 5000EUR from various income sources. Quartiles computed based on this approximated income | various sources of income (including wage, social security and social assistance payments) in brackets of 5000 EUR (KSZ) |
| **Unemployed** | **/** | / | / | / |
| **Self-employed** | **/** | / | / | / |

# Table S3 - Construction of employment quality indicators in CBSS

# Table S3 – Employment trajectory type and background characteristics among men. Source: CBSS

| **Employment trajectory cluster** | **Non-Belgian first nationality** |
| --- | --- |
| Standard | 14.66% |
| Income mobility | 26.03% |
| Multiple job-holder | 20.36% |
| High-earner | 10.44% |
| Slowly converging | 18.50% |
| Transitioning into self-employment | 16.02% |
| Modest income | 19.59% |
| Fluctuating high-income | 13.13% |
| Weaker attachment | 29.65% |
| Self-employed | 23.63% |
| Unstable | 44.57% |
| Unemployed | 46.32% |
| *Total* | *18.31%* |

# Table S4 – Employment trajectory type and background characteristics among women. Source: CBSS

| **Employment trajectory type** | **Non-Belgian first nationality** |
| --- | --- |
| Standard | 11.9% |
| Modest income standard | 15.5% |
| Favourable part-time | 8.5% |
| High-earner | 10.9% |
| Unstable | 25.1% |
| Unfavourable part-time | 16.6% |
| Low hours | 12.2% |
| Multiple job-holder | 19.0% |
| Transitioning into self-employment | 15.1% |
| Unemployed | 36.3% |
| *Total* | *14.3%* |

| ***Income*** | *Income mobility* | *Multiple job-holder* | *High-earner* | *Slowly converging* | *Transitioning into SE* | *Standard* | *Modest income* | *Fluctuating high-income* | *Weaker attachment* | *Self-employed* | *Unstable* | *Unemployed* |
| --- | --- | --- | --- | --- | --- | --- | --- | --- | --- | --- | --- | --- |
| *Lowest quartile* | 4 | 1.5 | 0 | 0 | 2.9 | 0 | 0 | 0 | 12.7 | 7 | 11.9 | 15.7 |
| *2^nd^ quartile* | 8.5 | 3.2 | 0 | 8.3 | 3.6 | 0.9 | 16 | 0 | 2.9 | 2.6 | 2.6 | 0.2 |
| *3^rd^ quartile* | 3 | 6.5 | 0.1 | 7.7 | 4.6 | 14.1 | 0 | 7.1 | 0.4 | 2.7 | 1.2 | 0 |
| *Highest quartile* | 0.5 | 4.9 | 15.9 | 0 | 4.9 | 1 | 0 | 8.9 | 0 | 3.6 | 0.3 | 0 |
| ***Multiple job-holding*** |  |  |  |  |  |  |  |  |  |  |  |  |
| *1 job* | 15.5 | 5.1 | 15.8 | 15.9 | 7.8 | 15.8 | 15.9 | 15.9 | 15.6 | 0.6 | 8.3 | 0.8 |
| *Multiple jobholding* | 0.1 | 10.8 | 0.2 | 0.1 | 0.2 | 0.1 | 0.1 | 0.1 | 0.1 | 0.1 | 0.1 | 0 |
| *Self-employment* | 0 | 0.1 | 0 | 0 | 7.9 | 0 | 0 | 0 | 0 | 15 | 0.5 | 0.1 |
| *Unemployment* | 0.4 | 0 | 0 | 0 | 0.1 | 0 | 0 | 0 | 0.3 | 0.3 | 7.1 | 15.2 |
| ***Employment stability*** |  |  |  |  |  |  |  |  |  |  |  |  |
| *Temporary/seasonal* | 0.6 | 0.2 | 0.1 | 0.1 | 0.2 | 0.3 | 0.1 | 0.1 | 0.3 | 0 | 1.1 | 0.2 |
| *Subsidised* | 0.4 | 0.2 | 0 | 0 | 0.1 | 0 | 0.1 | 0 | 1.1 | 0 | 2.4 | 0.3 |
| *Stable. no job change* | 14.2 | 14.3 | 15.6 | 15.5 | 7.5 | 15.4 | 15.6 | 15.7 | 14.1 | 0.6 | 4.7 | 0.3 |
| *Job change* | 0.4 | 1.2 | 0.3 | 0.3 | 0.3 | 0.3 | 0.2 | 0.2 | 0.3 | 0 | 0.2 | 0 |
| *Self-employment* | 0 | 0.1 | 0 | 0 | 7.9 | 0 | 0 | 0 | 0 | 15 | 0.5 | 0.1 |
| *Unemployment* | 0.4 | 0 | 0 | 0 | 0.1 | 0 | 0 | 0 | 0.3 | 0.3 | 7.1 | 15.2 |
| ***Working hours*** |  |  |  |  |  |  |  |  |  |  |  |  |
| *<21%* | 0 | 0.2 | 0 | 0 | 0 | 0 | 0 | 0 | 0 | 0 | 0 | 0 |
| *21-50%* | 0.1 | 0.4 | 0 | 0 | 0.2 | 0 | 0 | 0 | 1.8 | 0.1 | 0.7 | 0.1 |
| *51-80%* | 0.4 | 0.6 | 0.3 | 0 | 0.3 | 0.4 | 0 | 0.2 | 3.3 | 0.1 | 0.9 | 0.1 |
| *81-100%* | 15.1 | 6 | 15.5 | 15.9 | 7.4 | 15.4 | 15.9 | 15.7 | 10.6 | 0.5 | 6.7 | 0.5 |
| *100%<* | 0.1 | 8.6 | 0.1 | 0 | 0.1 | 0.1 | 0 | 0 | 0.1 | 0 | 0 | 0 |
| *Self-employment* | 0 | 0.1 | 0 | 0 | 7.9 | 0 | 0 | 0 | 0 | 15 | 0.5 | 0.1 |
| *Unemployment* | 0.4 | 0 | 0 | 0 | 0.1 | 0 | 0 | 0 | 0.3 | 0.3 | 7.1 | 15.2 |

# Table S5 - Average time (expressed in quarters) spent in each state by employment trajectory cluster among men between 2006 and 2009 (total=16). Source: CBSS

| ***Income*** | *Modest income standard* | *Favourable part-time* | *Standard* | *High-earner* | *Unstable* | *Unfavourable part-time* | *Low hours* | *Multiple job-holder* | *Transitioning into self-employment* | *Unemployed* |
| --- | --- | --- | --- | --- | --- | --- | --- | --- | --- | --- |
| *Lowest quartile* | 3.1 | 0.4 | 0.2 | 0 | 9.7 | 13.5 | 8.4 | 3.5 | 6.5 | 14.5 |
| *2^nd^ quartile* | 9 | 5.2 | 1.0 | 0.1 | 4.6 | 2.3 | 4.8 | 5.0 | 3.2 | 1.2 |
| *3^rd^ quartile* | 3.8 | 6.6 | 13.9 | 2.4 | 1.5 | 0.2 | 2.1 | 4.9 | 3.4 | 0.3 |
| *Highest quartile* | 0.1 | 3.8 | 0.9 | 13.5 | 0.2 | 0 | 0.6 | 2.6 | 2.9 | 0 |
| ***Multiple job-holding*** |  |  |  |  |  |  |  |  |  |  |
| *1 job* | 15.9 | 15.9 | 15.7 | 15.7 | 13.3 | 15.6 | 15.8 | 3.6 | 5.0 | 1.7 |
| *Multiple jobholding* | 0.1 | 0 | 0.3 | 0.2 | 0.4 | 0.3 | 0.2 | 12.2 | 0.2 | 0 |
| *Self-employment* | 0 | 0 | 0.1 | 0 | 0.1 | 0 | 0 | 0.1 | 10.3 | 0 |
| *Unemployment* | 0.1 | 0 | 0 | 0 | 2.3 | 0.1 | 0 | 0.1 | 0.5 | 14.2 |
| ***Employment stability*** |  |  |  |  |  |  |  |  |  |  |
| *Temporary/seasonal* | 0.2 | 0 | 0.1 | 0.1 | 1.2 | 0.2 | 0 | 0.3 | 0.1 | 0.2 |
| *Subsidised* | 0.1 | 0 | 0 | 0 | 8.1 | 0.8 | 0.1 | 1.6 | 0.3 | 0.9 |
| *Stable. no job change* | 15.4 | 15.8 | 15.5 | 15.6 | 4.2 | 14.8 | 15.6 | 13.1 | 4.6 | 0.6 |
| *Job change* | 0.2 | 0.1 | 0.2 | 0.2 | 0.2 | 0.3 | 0.1 | 0.8 | 0.1 | 0 |
| *Self-employment* | 0 | 0 | 0.1 | 0 | 0.1 | 0 | 0 | 0.1 | 10.3 | 0 |
| *Unemployment* | 0.1 | 0 | 0 | 0 | 2.3 | 0.1 | 0 | 0.1 | 0.5 | 14.2 |
| ***Working hours*** |  |  |  |  |  |  |  |  |  |  |
| *<21%* | 0 | 0 | 0 | 0 | 0.1 | 0.4 | 0.1 | 0.4 | 0 | 0 |
| *21-50%* | 0.3 | 0.4 | 0.3 | 0.1 | 4.3 | 1.0 | 13.6 | 0.8 | 0.6 | 0.4 |
| *51-80%* | 1.4 | 14.1 | 0.7 | 0.8 | 4.5 | 13.6 | 1.3 | 3.3 | 1.8 | 0.5 |
| *81-100%* | 14.2 | 1.5 | 14.8 | 14.9 | 4.5 | 0.9 | 1.0 | 5.7 | 2.7 | 0.8 |
| *100%<* | 0 | 0 | 0.1 | 0.1 | 0.1 | 0.1 | 0 | 5.7 | 0.1 | 0 |
| *Self-employment* | 0 | 0 | 0.1 | 0 | 0.1 | 0 | 0 | 0.1 | 10.3 | 0 |
| *Unemployment* | 0.1 | 0 | 0 | 0 | 2.3 | 0.1 | 0 | 0.1 | 0.5 | 14.2 |

# Table S6 - Average time spent (expressed in quarters) in each state by employment trajectory cluster among women between 2006 and 2009 (total=16). Source: CBSS

# Table S7 - Associations between type of employment trajectory 2006-2009 and disability from mental health disorder 2010-2016 among men. Hazard ratios (and 95% confidence intervals) from Cox proportional hazards regressions using interval censoring. Sensitivity analysis. Source: CBSS

|  | Adjusted regression estimates |
| --- | --- |
|  |  |
| Standard (ref.) | 1.00 |
|  |  |
| Income mobility | 2.77^***^ |
|  | (2.03,3.78) |
|  |  |
| Multiple job-holder | 1.51 |
|  | (0.72,3.16) |
|  |  |
| High-earner | 0.78 |
|  | (0.54,1.12) |
|  |  |
| Slowly converging | 1.63^**^ |
|  | (1.15,2.31) |
|  |  |
| Transitioning into self-employment | 1.17 |
|  | (0.66,2.06) |
|  |  |
| Modest income | 1.68^*^ |
|  | (1.12,2.51) |
|  |  |
| Fluctuating high-income | 1.12 |
|  | (0.72,1.74) |
|  |  |
| Weaker attachment | 3.06^***^ |
|  | (2.11,4.42) |
|  |  |
| Self-employed | 1.50 |
|  | (0.74,3.03) |
|  |  |
| Unstable | 7.02^***^ |
|  | (4.80,10.28) |
|  |  |
| Unemployed | 8.67^***^ |
|  | (6.03,12.46) |
|  |  |
| Belgian first nationality | 1.00 |
|  | (0.82,1.22) |
|  |  |
| Partner in HH | 0.61^***^ |
|  | (0.51,0.72) |
|  |  |
| Age | 1.03^*^ |
|  | (1.00,1.06) |
| N | 45 667 |

Exponentiated coefficients; 95% confidence intervals in brackets

^*^ p < 0.05, ^**^ p < 0.01, ^***^ p < 0.001

# Table S8- Associations between type of employment trajectory 2006-2009 and disability from mental health disorder 2010-2016 among women. Hazard ratios (and 95% confidence intervals) from Cox proportional hazards regressions using interval censoring. Sensitivity analysis. Source: CBSS

|  | Adjusted regression estimates |
| --- | --- |
|  |  |
| Standard (ref.) | 1.00 |
|  |  |
| Modest income standard | 1.43^**^ |
|  | (1.12,1.82) |
|  |  |
| Favourable part-time | 1.42^*^ |
|  | (1.08,1.87) |
|  |  |
| High-earner | 0.96 |
|  | (0.74,1.26) |
|  |  |
| Unstable | 2.95^***^ |
|  | (2.29,3.79) |
|  |  |
| Unfavourable part-time | 2.05^***^ |
|  | (1.54,2.73) |
|  |  |
| Low hours | 1.79^***^ |
|  | (1.32,2.42) |
|  |  |
| Multiple job-holder | 1.09 |
|  | (0.63,1.88) |
|  |  |
| Transitioning into SE | 1.60^*^ |
|  | (1.10,2.32) |
|  |  |
| Unemployed | 5.21^***^ |
|  | (3.97,6.84) |
|  |  |
| Belgian first nationality | 0.78^**^ |
|  | (0.67,0.90) |
|  |  |
| Partner in HH | 0.53^***^ |
|  | (0.46,0.60) |
|  |  |
| Age | 0.99 |
|  | (0.98,1.01) |
| N | 41 065 |

Exponentiated coefficients; 95% confidence intervals in brackets

^*^ p < 0.05, ^**^ p < 0.01, ^***^ p < 0.001

# Table S9 - Associations between employment trajectories and all-cause disability 2010-2016 among men. Sensitivity analyses. Source: CBSS

|  | Unadjusted regression estimates | Adjusted regression estimates |
| --- | --- | --- |
| Standard (ref) | 1.00 | 1.00 |
|  |  |  |
| Income mobility | 2.52^***^ | 2.53^***^ |
|  | (2.17,2.93) | (2.17,2.94) |
|  |  |  |
| Multiple job-holder | 1.00 | 1.00 |
|  | (0.65,1.54) | (0.65,1.53) |
|  |  |  |
| High-earner | 0.37^***^ | 0.37^***^ |
|  | (0.30,0.46) | (0.30,0.46) |
|  |  |  |
| Slowly converging | 1.53^***^ | 1.52^***^ |
|  | (1.29,1.81) | (1.28,1.79) |
|  |  |  |
| Transitioning into self-employment | 0.97 | 1.04 |
|  | (0.73,1.29) | (0.78,1.38) |
|  |  |  |
| Modest income | 1.67^***^ | 1.61^***^ |
|  | (1.37,2.02) | (1.33,1.96) |
|  |  |  |
| Fluctuating high-income | 0.84 | 0.83 |
|  | (0.67,1.06) | (0.66,1.05) |
|  |  |  |
| Weaker attachment | 2.25^***^ | 2.14^***^ |
|  | (1.85,2.73) | (1.76,2.61) |
|  |  |  |
| Self-employed | 1.21 | 1.24 |
|  | (0.84,1.75) | (0.86,1.79) |
|  |  |  |
| Unstable | 5.01^***^ | 4.72^***^ |
|  | (4.08,6.16) | (3.82,5.82) |
|  |  |  |
| Unemployed | 6.06^***^ | 5.21^***^ |
|  | (4.98,7.36) | (4.26,6.37) |
| Belgian first nationality |  | 0.92 |
|  |  | (0.83,1.02) |
|  |  |  |
| Partner in HH |  | 0.77^***^ |
|  |  | (0.70,0.84) |
|  |  |  |
| Age |  | 1.07^***^ |
|  |  | (1.06,1.09) |
| N | 45 667 | 45 667 |

Exponentiated coefficients; 95% confidence intervals in brackets

^*^ p < 0.05, ^**^ p < 0.01, ^***^ p < 0.001

# Table S10 - Associations between employment trajectories 2006-2009 and all-cause disability 2010-2016 among women. Sensitivity analyses. Source: CBSS.

|  | Unadjusted regression estimates | Adjusted regression estimates |
| --- | --- | --- |
| Standard (ref) | 1.00 | 1.00 |
| Modest income standard | 1.78^***^ | 1.76^***^ |
|  | (1.54,2.07) | (1.52,2.04) |
|  |  |  |
| Favourable part-time | 1.51^***^ | 1.56^***^ |
|  | (1.28,1.78) | (1.32,1.84) |
|  |  |  |
| High-earner | 0.81^*^ | 0.80^**^ |
|  | (0.68,0.96) | (0.67,0.95) |
|  |  |  |
| Unstable | 3.92^***^ | 3.58^***^ |
|  | (3.36,4.57) | (3.06,4.18) |
|  |  |  |
| Unfavourable part-time | 2.06^***^ | 2.08^***^ |
|  | (1.73,2.45) | (1.74,2.48) |
|  |  |  |
| Low hours | 1.87^***^ | 1.92^***^ |
|  | (1.56,2.23) | (1.60,2.29) |
|  |  |  |
| Multiple job-holder | 1.52^**^ | 1.45^*^ |
|  | (1.12,2.05) | (1.07,1.96) |
|  |  |  |
| Transitioning into self-employment | 1.69^***^ | 1.68^***^ |
|  | (1.34,2.13) | (1.34,2.12) |
|  |  |  |
| Unemployed | 6.03^***^ | 5.14^***^ |
|  | (5.07,7.16) | (4.31,6.13) |
|  |  |  |
| Belgian first nationality |  | 0.86^**^ |
|  |  | (0.78,0.94) |
|  |  |  |
| Partner in HH |  | 0.74^***^ |
|  |  | (0.68,0.80) |
|  |  |  |
| Age |  | 1.05^***^ |
|  |  | (1.04,1.06) |
| N | 41 065 | 41 065 |

Exponentiated coefficients; 95% confidence intervals in brackets

^*^ p < 0.05, ^**^ p < 0.01, ^***^ p < 0.001

# Table S11 - Associations between type of employment trajectory 2006-2009 and disability from mental health disorder 2010-2015 among men. Hazard ratios (and 95% confidence intervals) from Cox proportional hazards regressions. Sensitivity analysis. Source: CBSS

|  | Adjusted regression estimates |
| --- | --- |
| Standard (ref.) | 1.00 |
|  |  |
| Income mobility | 3.04^***^ |
|  | (2.11,4.37) |
|  |  |
| Multiple job-holder | 1.32 |
|  | (0.52,3.35) |
|  |  |
| High-earner | 0.80 |
|  | (0.52,1.23) |
|  |  |
| Slowly converging | 1.59^*^ |
|  | (1.05,2.41) |
|  |  |
| Transitioning into self-employment | 1.22 |
|  | (0.63,2.36) |
|  |  |
| Modest income | 1.65^*^ |
|  | (1.03,2.66) |
|  |  |
| Fluctuating high-income | 1.20 |
|  | (0.72,2.00) |
|  |  |
| Weaker attachment | 3.01^***^ |
|  | (1.94,4.67) |
|  |  |
| Self-employed | 1.41 |
|  | (0.60,3.31) |
|  |  |
| Unstable | 8.40^***^ |
|  | (5.46,12.92) |
|  |  |
| Unemployed | 9.34^***^ |
|  | (6.16,14.17) |
| Belgian first nationality | 1.04 |
|  | (0.83,1.30) |
|  |  |
|  |  |
| Partner in HH | 0.57^***^ |
|  | (0.47,0.69) |
|  |  |
| Age | 1.04^*^ |
|  | (1.01,1.07) |
| N | 45 667 |

Exponentiated coefficients; 95% confidence intervals in brackets

^*^ p < 0.05, ^**^ p < 0.01, ^***^ p < 0.001

# Table S12 - Associations between type of employment trajectory 2006-2009 and disability from mental health disorder 2010-2015 among women. Hazard ratios (and 95% confidence intervals) from Cox proportional hazards regressions. Sensitivity analysis. Source: CBSS

|  | Adjusted regression estimates |
| --- | --- |
| Standard (ref.) | 1.00 |
| Modest income standard | 1.55^**^ |
|  | (1.18,2.04) |
|  |  |
| Favourable part-time | 1.31 |
|  | (0.95,1.81) |
|  |  |
| High-earner | 0.98 |
|  | (0.72,1.33) |
|  |  |
| Unstable | 3.25^***^ |
|  | (2.44,4.31) |
|  |  |
| Unfavourable part-time | 2.01^***^ |
|  | (1.45,2.79) |
|  |  |
| Low hours | 1.70^**^ |
|  | (1.20,2.41) |
|  |  |
| Multiple job-holder | 1.06 |
|  | (0.56,1.99) |
|  |  |
| Transitioning into self-employment | 1.79^**^ |
|  | (1.19,2.71) |
|  |  |
| Unemployed | 5.79^***^ |
|  | (4.27,7.85) |
|  |  |
| Belgian first nationality | 0.82^*^ |
|  | (0.69,0.97) |
|  |  |
| Partner in HH | 0.54^***^ |
|  | (0.47,0.63) |
|  |  |
| Age | 1.01 |
|  | (0.99,1.03) |
| N | 41 065 |

Exponentiated coefficients; 95% confidence intervals in brackets

^*^ p < 0.05, ^**^ p < 0.01, ^***^ p < 0.001
